# Porous Carbon Substrate Improving the Sensing Performance of Copper Nanoparticles Toward Glucose

**DOI:** 10.1186/s11671-021-03579-y

**Published:** 2021-08-06

**Authors:** Zewen Qu, Shi Li, Wenshuai Feng, Shuting Kan, Xiaohui Gao, Aimin Guo, Hongjian Li, Lianwen Deng, Shengxiang Huang, Yan Zhao, Wei Chen

**Affiliations:** 1 School of Physics and Electronics, Hunan Key Laboratory for Super-Microstructure and Ultrafast Process, Central South University, Changsha, 410083 Hunan China; 2 College of Chemistry and Chemical Engineering, Central South University, Changsha, 410083 Hunan China; 3 State Key Laboratory of Electroanalytical Chemistry, Changchun Institute of Applied Chemistry, Chinese Academy of Sciences, Changchun, 130022 China; 4 Niversity of Science and Technology of China, Hefei, 230029 Anhui China

**Keywords:** Sensor, Copper nanoparticles, Glucose detection, Electrochemistry

## Abstract

An accurate sensor to rapidly determine the glucose concentration is of significant importance for the human body health, as diabetes has become a very high incidence around the world. In this work, copper nanoparticles accommodated in porous carbon substrates (Cu NP@PC), synthesized by calcinating the filter papers impregnated with copper ions at high temperature, were designed as the electrode active materials for electrochemical sensing of glucose. During the formation of porous carbon, the copper nanoparticles spontaneously accommodated into the formed voids and constituted the half-covered composites. For the electrochemical glucose oxidation, the prepared Cu NP@PC composites exhibit much superior catalytic activity with the current density of 0.31 mA/cm^2^ at the potential of 0.55 V in the presence of 0.2 mM glucose. Based on the high electrochemical oxidation activity, the present Cu NP@PC composites also exhibit a superior glucose sensing performance. The sensitivity is determined to be 84.5 μA /(mmol^.^L) with a linear range of 0.01 ~ 1.1 mM and a low detection limit (LOD) of 2.1 μmol/L. Compared to that of non-porous carbon supported copper nanoparticles (Cu NP/C), this can be reasonable by the improved mass transfer and strengthened synergistic effect between copper nanoparticles and porous carbon substrates.

**Supplementary Information:**

The online version contains supplementary material available at 10.1186/s11671-021-03579-y.

## Introduction

In recent years, diabetes has raised great attention worldwide, promoting the rapid and accurate determination for glucose concentration [1]. Various techniques have been developed [2]. With the merits of easy operation, fast response and high sensitivity, electrochemical methods are of particular interest in glucose sensing, and the electrode active materials are of utmost importance for the sensors [3, 4]. So far, the reported materials with good glucose response activity include noble metals (gold [4], silver [5], platinum [6], palladium [7]), non-noble metal (copper [8], nickel [9]), metal oxides (zinc oxide [10], manganese oxide [11], nickel oxide [12], iron oxide [13]), and carbon materials (carbon nanotubes [14], carbon nanodots [15], mesoporous carbon [16]), etc. Among these materials, copper-based composites show the great potential for constructing an efficient sensing platform for glucose, as a result of the low cost [3], good electrical conductivity [17], controlled specific surface area. Meanwhile, it is reported that the electrochemical performance of copper-based materials will be significantly improved by forming composites with carbonaceous substrates such as grapheme [18, 19], carbon nanofibers [20], carbon nanotubes [21] and mesoporous carbons [22]. For example, Zhang et al. prepared the copper nanoparticles on laser-induced graphene composites and successfully developed a flexible enzyme-free glucose amperometric biosensor. Benefiting from its simplicity and high sensitivity, the sensor was expected to be used in wearable or implantable biosensors [23]. Using arc discharge method, the composite materials of CuO and single-wall carbon nanotubes were synthesized by Wang’s group. The highly conductive network facilitated by carbon nanotubes leaded to high sensitivity and good selectivity in glucose sensing [21]. Because of the good conductivity of copper nanowires and fast electron transfer in two-dimensional reduced graphene oxide (rGO) layers, Ju et al. synthesized a composite of one-dimensional copper nanowires and two-dimensional rGO nanosheets, showing a sensitivity of 1625 $\upmu$A/(mM·cm^2^) and a limit detection of 0.2 $\upmu$M for the detection of glucose [3]. The much performance enhancement of copper-based materials have achieved, however, it is still not enough for the real applications of portable device. This means that it is necessary to search new templates or matches for copper nanoparticles.

With the special three-dimensional framework structure [24], porous carbons not only possess abundant binding sites to promote the dispersion of metal active centers, but also provide a larger specific surface area that improves the accessibility of electrons and reactive substances [25–27]. In recent years, porous carbons have been recognized as a type of promising modification and substrate materials, which can greatly enhance the electrochemical sensing activity of metal materials. For instance, Li et al. investigated the composites of Co_7_Fe_3_ alloy nanoparticles embedded in porous carbon nanosheets (Co_7_Fe_3_/NPCSs). The results showed a much wide linear range for the detection of glucose (from 0.001 to 14.00 mM), due to the nanoconfined effect from the porous carbon [28]. Using the metal–organic frameworks (MOFs) as self-sacrificial templates to prepare porous carbon materials, the nickel nanoparticles embedded on nanoporous carbon nanorods prepared by Jia et al. presented good glucose sensing properties with the fast response times (within 1.6 s) [29]. Song et al. constructed a composite (Cu@C-500) consisting of copper nanoparticle uniformly embedded porous carbon bed by using Cu MOF as a raw material. Because of the hierarchical porosity, it exhibited high sensitivity and low detection limit, and presented great potential in glucose sensor devices [30]. Therefore, with the unique structural and electronic effects, porous carbon material is anticipated to be an excellent partner for further enhancing the electrochemical performance of copper nanomaterials in the glucose sensing.

Herein, in this work, the composites of copper nanoparticles accommodated in porous carbon substrates were designed and synthesized by calcinating the cheap filter papers impregnated with copper ions at high temperature. During the synthesized process, the formation of porous carbon and the accommodation of copper nanoparticles simultaneously occurred, which can be demonstrated by scanning electron microscopy and transmission electron microscopy. For the electrochemical measurements, the results show that the prepared samples (Cu NP@PC) exhibit high electrocatalytic activity for glucose oxidation with the current density of 0.31 mA/cm^−2^ at the potential of 0.55 V in the presence of 0.2 mM glucose, which is much better than that from the Cu NP/C. For the glucose sensing, the sensitivity is determined to be 84.5 μA (mmol/L)^−1^ and the detection limit is calculated to be 2.1 μmol/L, much superior to those from most of the previously reported materials. Furthermore, the good selectivity of present materials was also demonstrated by the anti-interference experiment.

## Experimental

### Reagents

Copper nitrate (Cu(NO_3_)_2_·3H_2_O, AR), ethanol (C_2_H_5_OH, 99.8%), glucose (C_6_H_12_O_6_, 96%), urea (CH_4_N_2_O, AR, 99%), citric acid (C_6_H_8_O_7_, AR, 99.5%), ammonium acetate (CH_3_COONH_4_, AR), sodium chloride (NaCl, AR, 99.5%), potassium hydroxide (KOH, AR, 85%). All the reagents mentioned above were purchased from Aladdin. The 5% D520 Nafion solution obtained from DuPont, and the filter paper was purchased from Hangzhou Fuyang BEIMU Pulp Co., Ltd. Carbon paper from Japan's Toray conductive carbon paper (TGP-060). The water used in the whole experiment is ultrapure with the conductivity of 18.25 MΩ⋅cm.

### Instruments

X-ray diffraction (XRD) spectra were obtained from instrument X’Pert PRO MPD multi-purpose powder X-ray diffractometer. Fourier transform infrared spectra (FT-IR) in the range of 1000–4000 cm^−1^ were recorded from the IS50 FT-IR spectrometer. Raman spectra were measured in the inVia Qontor (Renishaw, UK) system at a wavelength of 532 nm. X-ray photoelectron spectroscopy (XPS) measurements were performed on a Thermo ESCALAB 250XI spectrometer running at 120 W. The morphologies of the sample were characterized by Hitachi S4800 scanning electron microscope (SEM) with a working accelerating voltage of 20 kV. The transmission electron microscopy (TEM) images were collected from the Tecnai G2 F20. The Brunauer–Emmett–Teller (BET) measurements were performed on the specific surface area physical adsorption apparatus (ASAP2020M).

### Synthesis of Cu NP@PC and Cu NP/C

Typically, the synthesis of Cu NP@PC was completed by two-step high temperature pyrolysis. First, the commercial filter papers were allowed to pre-treat at 250 °C for 1 h in a tube furnace under nitrogen atmosphere. Next, a piece of treated pale yellow filter paper with the size of 10 mm $\times$ 50 mm was soaked in blue transparent copper nitrate solution with a concentration of 0.1 M, and was taken out after 10 min. After drying at room temperature, the filter paper was put into a clean porcelain boat and successively treated at 180 °C, 240 °C, 900 °C for 2 h, 2 h, and 1 h in a tubular furnace under nitrogen protection, respectively. Finally, the Cu NP@PC product was collected when the system was cooled to room temperature, and was grinded before electrochemical tests. For the control samples, the synthesis of Cu NP/C and pure carbon were carried out through the same procedure, except that the concentration of copper nitrate was 0.2 M and 0 M, respectively.

### Electrochemical Measurements

In this work, all electrochemical tests were performed on CHI 760E electrochemical workstation with a standard three-electrode system at room temperature. Before the experiment, several pieces of carbon papers (5 mm × 5 mm) as current collectors were rinsed with water, ethanol and dried overnight at 60 °C. For the preparation of catalyst ink, 10 mg sample (Cu NP@PC, Cu NP/C or pure carbon powders) was mixed with ethanol, water, and Nafion (5%) solution in a certain proportion of 10:10:1 to form a uniformly dispersion. Then, the catalyst ink of 40 μL was dropped on a clean carbon paper with a load of 1.6 mg/cm^2^, which was used as a working electrode. An Ag/AgCl (saturated KCl) electrode and a graphite rod were used as reference electrode and counter electrode, respectively. For the electrochemical experiments, cyclic voltammetry and linear sweep voltammetry were adopted to qualitatively examine the potential performance of the prepared material for glucose oxidation. The chronoamperometry was used to quantitatively evaluate the sensing performance of the prepared material. In the whole process, 0.1 M KOH solution was selected as the electrolyte.

## Results and Discussion

**Fig. 1:**
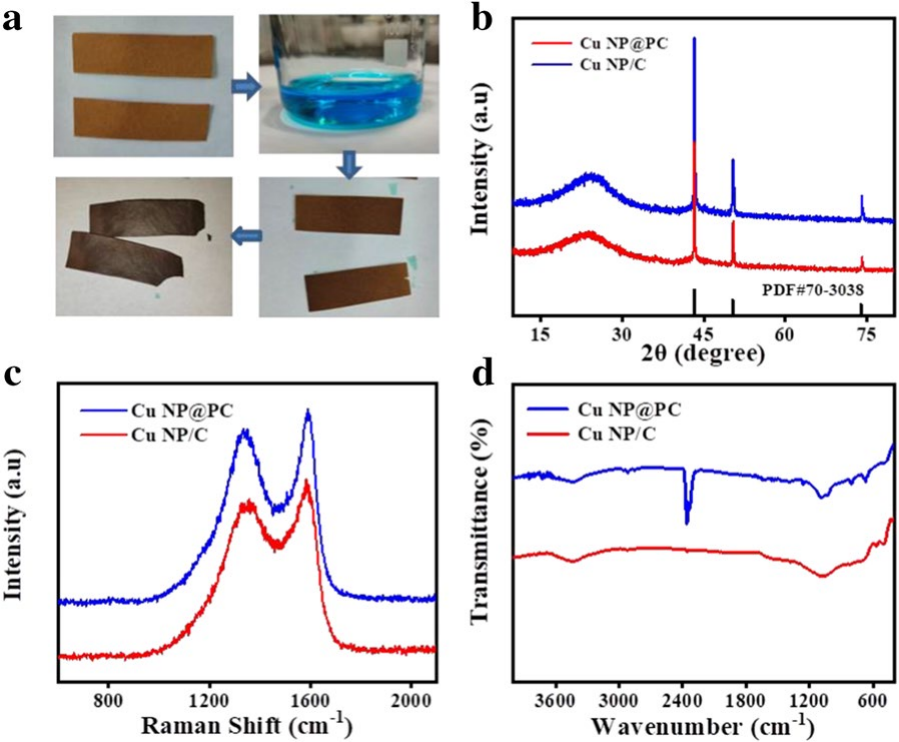
**a** Schematic illustration of preparation of samples of Cu NP@PC and Cu NP/C; **b** The X-ray diffraction (XRD) pattern of Cu NP@PC and Cu NP/C; **c** Raman spectra of Cu NP@PC and Cu NP/C; and **d** FTIR spectra of Cu NP@PC and Cu NP/C

As shown in Fig. 1a, for the synthesis of target materials, the preheating treatment was allowed to remove the unstable impurities and moisture from the filter paper with the color changing to light yellow. Then, to support the metal nanoparticles, the treated filter papers were infiltrated into the copper ion solution. During the high temperature calcination process in a tubular furnace, the copper atoms and tiny crystallites were formed. Because the nucleation and growth rate of copper nanoparticles is less than the pyrolysis rate of carbon, these initial copper microcrystals can catalyze the decomposition and evaporation of carbon, leading to the formation of holes [31]. Finally, the brown-black Cu NP@PC samples were prepared. Note that the excessive concentration of copper ions will increase the nucleation rate, causing the formation of non-porous carbon materials. To identify the components of the prepared sample, X-ray diffraction (XRD) patterns were collected, as shown in Fig. 1b. Both Cu NP@PC and Cu NP/C samples present the diffraction peaks of copper and carbon. The three sharp characteristic peaks located at diffraction angles of 43.2°, 50.3°, and 73.9° can be respectively attributed to the lattice planes of (111), (200) and (220) from the copper nanoparticles (PDF#04-0836) [32, 33]. The broad peak with the center around 25° corresponds to the (002) crystalline face from the graphitized carbon, which will promotes the electron transport in subsequent electrochemical reactions [3, 25, 34]. To analyze the specific compositions of carbon, the Raman spectra of Cu NP@PC and Cu NP/C were collected. As shown in Fig. 1c, the D-band and G-band can be unambiguously determined by the peak around 1350 cm^−1^ and 1600 cm^−1^, respectively [35]. As reported, the G band is caused by the relative motion of *sp*^2^ carbon atoms, while the D band is connected with the breathing mode of carbon rings [36]. Herein, the calculated D/G band ratio of Cu NP@PC was 0.899, the same with the value from Cu NP/C. Therefore, the distribution of amorphous carbon and nanocrystalline graphite are similar in two samples. This indicates the almost same components of two prepared materials, i.e., that both Cu NP@PC and Cu NP/C are consisted of copper nanoparticles and carbon frameworks. To further reveal the microstructure information, the FTIR spectra of Cu NP@PC and Cu NP/C were investigated. As presented in Fig. 1d, it can be seen that the signals located at 1734 cm^−1^ and 1628 cm^−1^ appear in Cu NP@PC which can be attributed to the stretching vibration of C=O [39] and the stretching vibration of C–O [40]. Compared to the Cu NP/C, the band at 2363 cm^−1^ from the Cu NP@PC is attributed to carbon dioxide in the air. A slight absorption band was observed at 3466 cm^−1^ from the Cu NP@PC and Cu NP/C could be assigned to O–H bond stretching vibration in molecule of water [37].

**Fig. 2:**
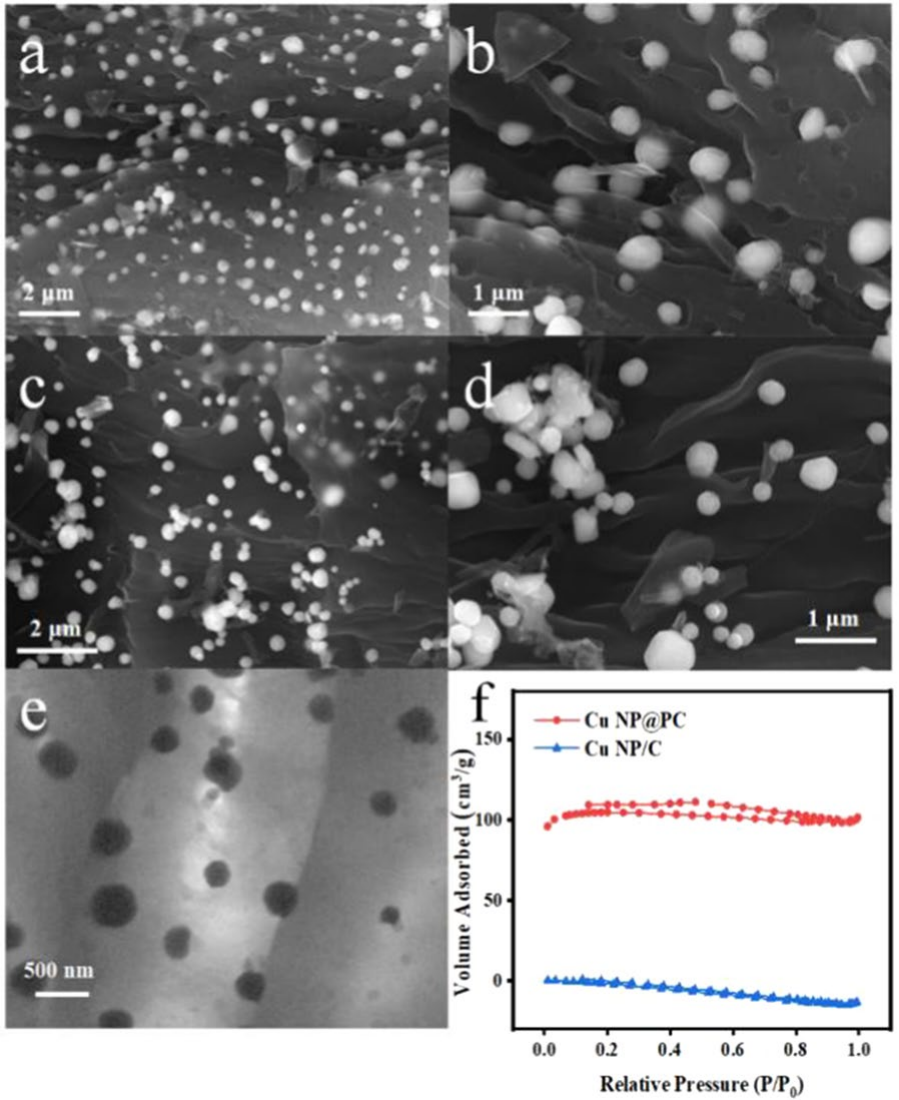
**a**, **b** The scanning electron microscope (SEM) images of Cu NP@PC at different magnifications; **c**, **d** The scanning electron microscope (SEM) images of Cu NP/C at different magnifications; **e** The transmission electron microscopy (TEM) image of Cu NP@PC; and **f** Brunauer–Emmett–Teller (BET) surface area analysis of Cu NP@PC and Cu NP/C

To observe the morphologies and structures of the prepared materials, the scanning electron microscope (SEM) experiments were conducted. For the Cu NP@PC sample, the SEM image in Fig. 2a shows that abundant holes are randomly distributed on the surface of carbon layer, and the copper nanoparticles just reside in these holes. Figure 2b presents that almost all copper nanoparticles are half inside and half outside. As it is reported, the electrochemical reaction usually involves electron and mass transport. Thus, the half inside will be conducive to electron transfer with the carbon substrate, while half outside can act as active sites, interacting with substances. This will ultimately improve the efficiency of electrochemical reactions. In Fig. 2c, no porous carbon was found and all the copper nanoparticles are supported on the surface of carbon in the Cu NP/C sample. Some agglomerations even occurred in Fig. 2d. In addition, the copper nanoparticles size from two samples was 0.406 and 0.398 μm, respectively, based on a hundred metal nanoparticles. Thus, the size of the copper nanoparticles grown under two different copper ion concentrations is not much different, indicating that increasing copper ion concentration can only control the morphology of the carbon. Moreover, it can be seen from the TEM image in Fig. 2e that the enlarged copper nanoparticles have a size similar to these holes and partially encapsulated in them, again indicating that the successful formation of the target composites. To further reveal the porous properties of prepared materials, the nitrogen adsorption isotherms of Cu NP@PC and Cu NP/C were studied. As shown in Fig. 2f, the calculated BET surface area of the Cu NP@PC nanomaterials was 309.95 m^2^/g, much higher than that of Cu NP/C. This is consistent with the results from the SEM and TEM.

**Fig. 3:**
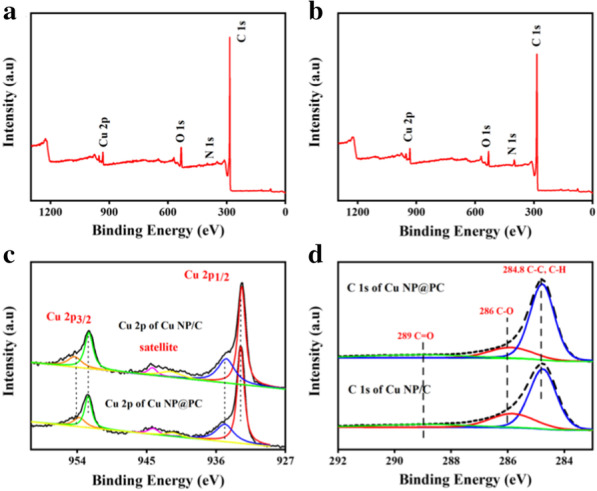
**a** XPS survey spectrum of Cu NP@PC; **b** XPS survey spectrum of Cu NP/C; **c** Cu 2*p* XPS spectra of Cu NP@PC and Cu NP/C; and **d** C 1 XPS spectra of Cu NP@PC and Cu NP/C

To investigate the electronic structure of the samples, X-ray photoelectron spectroscopy (XPS) was carried out. Figure 3a and b display the full XPS survey spectra of Cu NP@PC and Cu NP/C, respectively, which show the existence of Cu, C and O. For the Cu element, Fig. 3c presents the deconvoluted Cu 2*p* XPS spectra of the Cu NP@PC and Cu NP/C. Both signals were produced at the same peak positions, hinting the same composition of two samples. Two obvious peaks at 952.5 eV and 932.8 eV are attributed to the Cu 2*p*_3/2_ and Cu 2*p*_1/2_ of Cu (0), suggesting the presence of metal copper [38]. The binding energies at 953.7 eV and at 934.8 eV are assigned to the Cu 2*p*_3/2_ and Cu 2*p*_1/2_ from the Cu(II)[39–41]. The presence of Cu(II) can be also confirmed by weak satellite peaks at 944.2 eV and 941.4 eV[10]. From the fitting peaks corresponding to Cu(0) and Cu(II), the ratios of Cu(0)/Cu(II) in Cu NP@PC and Cu NP/C are estimated to be 2.2 and 1.8, respectively. This can be explained by the fact that the surface copper atoms in Cu NP@PC are not easy to be oxidized due to the encapsulation of porous carbon layer. Meanwhile, more metal copper atoms may play an important role for the glucose sensing. For the C1*s* spectrum of the two samples in Fig. 3d, three signals located at 289 eV, 286 eV and 284.8 eV correspond to the C=O, C–O, C–C/C–H, respectively, indicating the existence of oxygen-containing functional groups such as carboxyl group [42, 43] and in consistent with the results from FTIR.

**Fig. 4:**
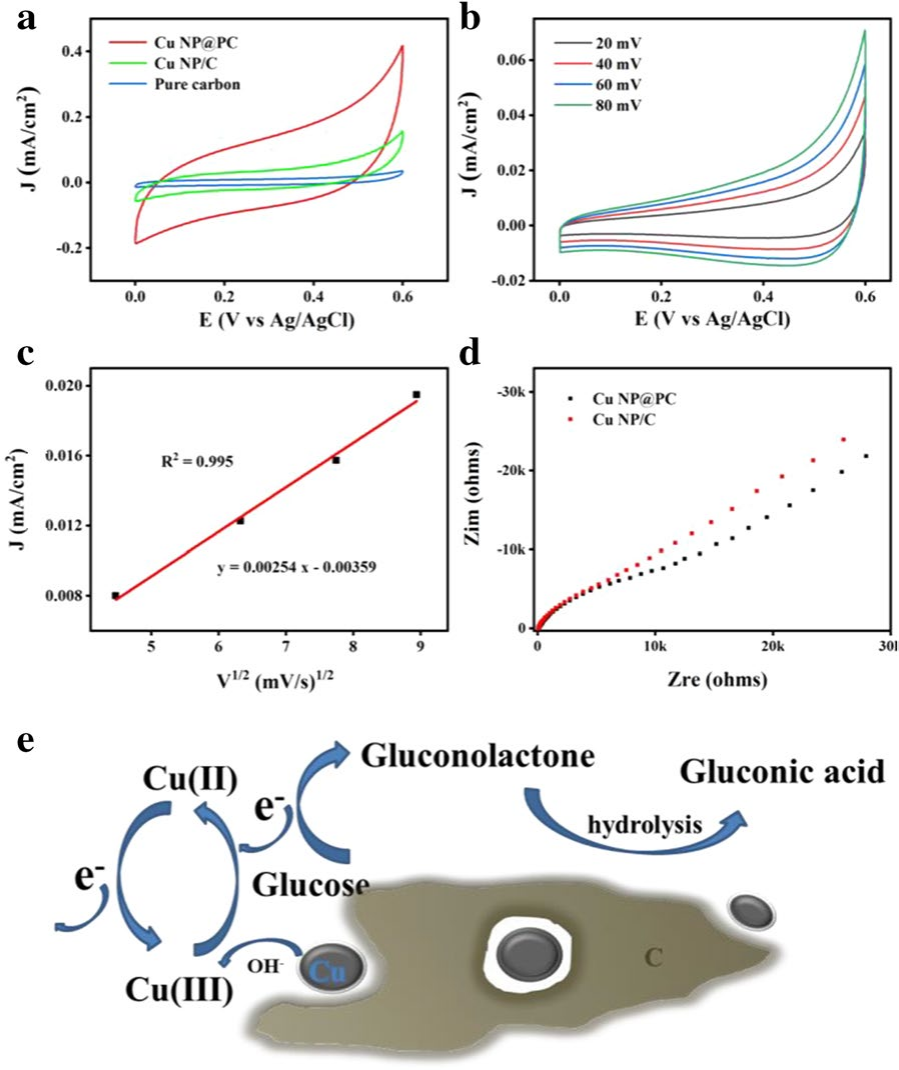
**a** CV curves of Cu NP@PC, Cu NP/C and pure carbon sample for glucose oxidation reaction; (0.2 mM glucose, 0.1 M KOH, scan rate: 100 mV/s.) **b** CV curves of Cu NP@PC in 0.1 M KOH at different scan rates (20, 40, 60, 80 mV/s); **c** Plot of current density at 0.4 V in relation to the square root of scan rate; **d** The electrochemical impedance spectra of Cu NP@PC and Cu NP/C; and **e** The schematic diagram of the mechanism process of glucose conversion on the Cu NP@PC

Based on the advantages of porous carbon, the electrochemical sensing properties of Cu NP@PC and Cu NP/C toward glucose were investigated in 0.1 M KOH solution. The pure carbon material without copper nanoparticles is used as the reference sample. As shown in Fig. 4a, the cyclic voltammetric curves (CV) show the largest current response from Cu NP@PC with the presence of 0.2 mM glucose in electrolyte, when compared to that of Cu NP/C and pure carbon sample. Specifically, the current density of 0.31 mA/cm^−2^ was obtained at the potential of 0.55 V. This indicates that the prepared Cu NP@PC is the best catalyst for glucose oxidation, which can be reasonable by its owning porous structure. As it is reported, the porosity can promote the mass transport [29]. Herein, to demonstrate the enhanced mass transport, the effect of scanning rates on the glucose oxidation was investigated on Cu NP@PC modified electrode. As shown in Fig. 4b, the current density increases in a gradient with the scanning rate changing from 20, 40, 60 to 80 mV/s. Figure 4c shows the fitting curve between the current density (*J*_p_) and the square root of scanning rate (*v*^1/2^). The linear relationship can be expressed as: *J*_p_ = 0.00254 *v*^1/2^ − 0.00359 (correlation coefficient: *R*^2^ = 0.995), indicating a diffusion controlled process of the glucose oxidation on the Cu NP@PC modified electrode [44]. Furthermore, in Fig. 4d, the electrochemical impedance spectra (EIS) present that the charge transfer resistance of Cu NP@PC is lower than that of Cu NP/C. Therefore, combining the promoted mass transport and the enhanced electron transfer process, the catalytic oxidation of glucose on the Cu NP@PC modified electrode can be sketched in Fig. 4e. The Cu(II) was first oxidized to Cu(III), which subsequently accepted an electron and was reduced to Cu(II). During this process, the glucose molecule donated an electron and was oxidized to gluconolactone. Benefiting from the materials’ porosity, the formed gluconolactone can be rapidly transfered into the solution and then hydrolyzed to gluconic acid [3, 45].

**Fig. 5:**
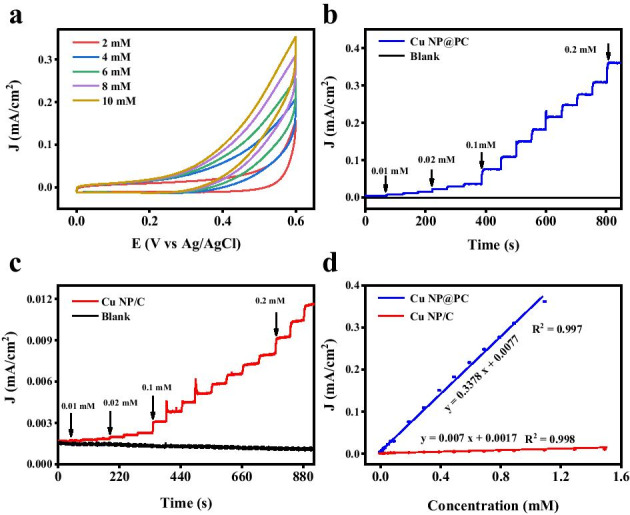
**a** CV curves of Cu NP@PC in 0.1 M KOH with the presence of glucose at different concentrations of 2, 4, 6, 8 and 10 mM. Scan rate: 100 mV/s; **b** The amperometric responses of Cu NP@PC upon successive addition of glucose solution in 0.1 M KOH at 0.55 V (vs Ag/AgCl); **c** The amperometric responses of Cu NP/C upon successive addition of glucose solution in 0.1 M KOH at 0.55 V (vs Ag/AgCl); and **d** The corresponding calibration curves of the Cu NP@PC and Cu NP/C for glucose sensing. The error bars were obtained based on three repeats of the experiment

**Fig. 6:**
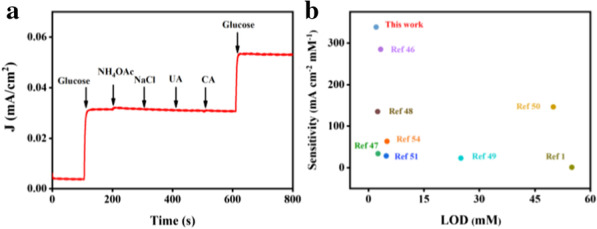
**a** The response currents of Cu NP@PC after injection of 0.01 mM glucose solution, 0.01 mM ammonium acetate (NH_4_OAc), 0.01 mM sodium chloride (NaCl), 0.01 mM urea (UA), 0.01 mM citric acid (CA), respectively; and **b** The compassion of LOD and sensitivity between Cu NP@PC and previously reported materials

According to the superior electrochemical catalytic oxidation performance, the potential sensing performance of Cu NP@PC toward glucose was examined. To qualitatively study the current response of Cu NP@PC toward glucose concentration, cyclic voltammetry was carried out in the concentrations of 2, 4, 6, 8 and 10 mM. As shown in Fig. 5a, the current density from the Cu NP@PC-modified electrode gradually increases with the glucose concentration increasing, hinting the potential excellent sensing performance. For quantificationally revealing the glucose sensing properties of Cu NP@PC, the chronoamperometry (I-t) was performed and the potential of 0.55 V was chosen. As shown in Fig. 5b, the current density from Cu NP@PC-modified electrode increases step by step with the glucose concentration increasing from 0.01 to 1.1 mM. From the I–t curves, in Fig. 5d, the fitted calibration curve between glucose concentrations and response currents can be expressed as: *y* = 0.3378 *x* + 0.0077 (correlation coefficient: *R*^2^ = 0.997). Meanwhile, the sensitivity was determined to be 84.5 μA (mmol/L)^−1^. According to the formula of LOD = 3*σ*/*q* [46] (σ refers to the standard deviation of the blank response and q is the slope of that linear regression curve), the detection limit was calculated to be 2.1 μmol/L. These two indexes are much better than those from most of the previous reports, as shown in Fig. 6b [47–52]. As comparison, the current density of I-t curve from Cu NP/C-modified electrode also shows a gradient change with the glucose concentration increasing, as shown in Fig. 5c. However, the magnitude of change was significantly reduced. As shown in Fig. 5d, the fitting linear curve between glucose concentrations and response current was represented as: *y* = 0.007 *x* + 0.0017 (correlation coefficient *R*^2^ = 0.998). The sensitivity was 1.75 μA (mmol/L)^−1^ and the detection limit was estimated to be 10 μmol/L. Therefore, compared to the results of Cu NP/C, the sensing performance of Cu NP@PC sample was also improved by the porous carbon substrate.

As is well-known, anti-interference ability is another key factor to evaluate the materials’ sensing performance. In this work, to investigate the selectivity of Cu NP@PC-modified electrode toward glucose, several interfering substances including ammonium acetate (NH_4_OAc), sodium chloride (NaCl), urea (UA), citric acid (CA) with the concentration of 0.01 mM were chosen and was injected successively into the electrolyte [53]. Obviously, the current density changes caused by the interfering substances can be negligible. Only when 0.01 mM glucose was injected, the current density increased significantly regardless of the above interferences, as shown in Fig. 6a. Moreover, using the urine as the substrate, this proposed system can still achieve the sensitivity detection of glucose, comparable to the commercial test paper (Additional file 1: Figure S3 and S4). Therefore, the Cu NP@PC materials possess an excellent electrochemical catalytic oxidation and sensing ability toward glucose.

## Conclusion

A composite consisting of copper nanoparticles and porous carbon substrates was designed and synthesized by calcinating the commercial filter papers impregnating with the copper ions. With the advantages of the porosity, the prepared Cu NP@PC showed an excellent ability for the electrochemical glucose oxidation and sensing. The sensitivity was determined to be 84.5 μA mM^−1^ and the limit of detection was calculated to be 2.1 μM, which is much superior to those from most of the previous reports. Furthermore, the Cu NP@PC-modified electrode also exhibited good selectivity for glucose. Therefore, the composite prepared in this work will provide not only a new candidate for constructing portable glucose sensors, but also a new thought for the preparation of porous carbon materials.

## Supplementary Information

**Additional file 1**. **Table S1.**the element content in the prepared Cu NP@PC before electrochemical test. **Table S2.** the element content in the prepared Cu NP@PC after electrochemical test. **Figure S3.** the amperometric responses of Cu NP@PC upon succesive addition of glucose urine solution in 0.1 M KOH. **Figure S4.** the results from commercial test paper at the concentration of 2.8 mM.

## Data Availability

The data and conclusions in this work are all showed in this paper.

## Abbreviations

LOD: Limit of detection
rGO: Reduced graphene oxide
MOF: Metal-organic framework
XRD: X-ray diffraction
SEM: Scanning electron microscope
TEM: Transmission electron microscopy
BET: Brunauer–Emmett–Teller
XPS: X-ray photoelectron spectroscopy
CV: Cyclic voltammetric curves
I-t: Chronoamperometry
UA: Urea
CA: Citric acid
