# Supplementary material for: Porous Carbon Substrate Improving the Sensing Performance of Copper Nanoparticles Toward Glucose
Source: Nanoscale Res Lett. 2021 Aug 6;16:127. doi: 10.1186/s11671-021-03579-y (PMC8346618; doi:10.1186/s11671-021-03579-y)
Supplement: Supplementary file 1 — Additional file 1. Table S1.the element content in the prepared Cu NP@PC before electrochemical test. Table S2. the element content in the prepared Cu NP@PC after electrochemical test. Figure S3. the amperometric responses of Cu NP@PC upon succesive addition of glucose urine solution in 0.1 M KOH. Figure S4. the results from commercial test paper at the concentration of 2.8 mM. [file 11671_2021_3579_MOESM1_ESM.docx]

Supporting Information

**Porous carbon substrate improving the sensing performance of copper nanoparticles toward glucose**

Zewen Qu^1^, Shi Li^1^, Wenshuai Feng^1^, Shuting Kan^2^, Xiaohui Gao^1^*^†^* (**🖂**), Aimin Guo^1^, Hongjian Li^1^, Lianwen Deng^1^, Shengxiang Huang^1^, Yan Zhao^1^, Wei Chen^1,3, 4^


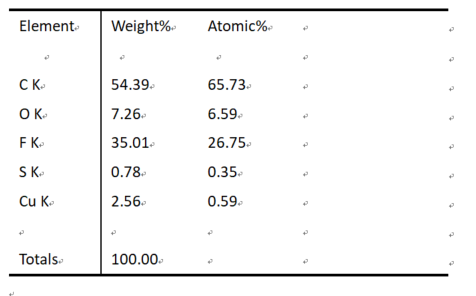


Table S1. the element content in the prepared Cu NP@PC before electrochemical test.


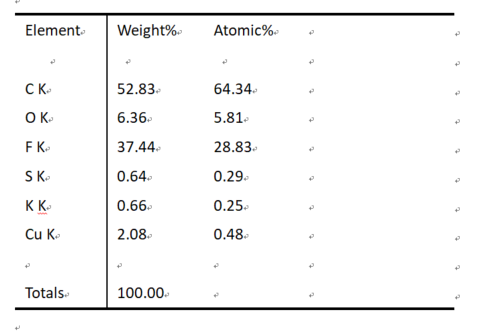


Table S2. the element content in the prepared Cu NP@PC after electrochemical test.

In Table 1 and 2, the mass percentage of copper was estimated to be 2.56 % and 2.08 %, respectively. This result indicates the copper content did not decrease significantly, and the slight change may be caused by the adsorption of potassium hydroxide. Therefore, the prepared Cu NP@PC materials exhibited the superior stability for electrochemical glucose sensing and will be a promising candidate for the construction of sensors.


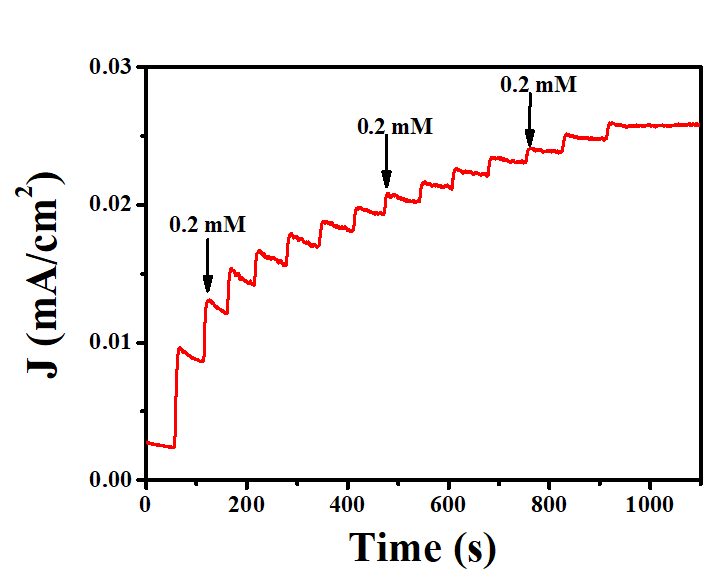


Figure S1. the amperometric responses of Cu NP@PC upon successive addition of glucose urine solution in 0.1 M KOH.


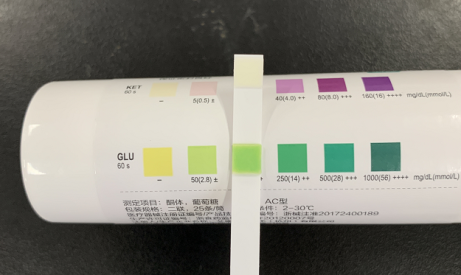


Figure S2. the results from commercial test paper at the concentration of 2.8 mM.

To investigate the real application of present materials, urine was chosen as the liquid substrate. As shown in Figure S1, the current density increases with the addition of glucose, indicating the good sensing capability of Cu NP@PC materials. Testing with the concentration of 2.8 mM, the result was comparable to that from commercial test paper, as shown in Figure S2. Since the corresponding portable device is being designed in our lab, these detail pictures were not presented for this time.
